# A Framework for Enabling Unpaired Multi-Modal Learning for Deep Cross-Modal Hashing Retrieval

**DOI:** 10.3390/jimaging8120328

**Published:** 2022-12-15

**Authors:** Mikel Williams-Lekuona, Georgina Cosma, Iain Phillips

**Affiliations:** Department of Computer Science, School of Science, Loughborough University, Loughborough LE11 3TT, UK

**Keywords:** hashing network, multi-modal deep learning, information retrieval, cross-modal

## Abstract

Cross-Modal Hashing (CMH) retrieval methods have garnered increasing attention within the information retrieval research community due to their capability to deal with large amounts of data thanks to the computational efficiency of hash-based methods. To date, the focus of cross-modal hashing methods has been on training with paired data. Paired data refers to samples with one-to-one correspondence across modalities, e.g., image and text pairs where the text sample describes the image. However, real-world applications produce unpaired data that cannot be utilised by most current CMH methods during the training process. Models that can learn from unpaired data are crucial for real-world applications such as cross-modal neural information retrieval where paired data is limited or not available to train the model. This paper provides (1) an overview of the CMH methods when applied to unpaired datasets, (2) proposes a framework that enables pairwise-constrained CMH methods to train with unpaired samples, and (3) evaluates the performance of state-of-the-art CMH methods across different pairing scenarios.

## 1. Introduction

Information retrieval refers to obtaining relevant information from a dataset when prompted by a query. When a dataset comprises samples from different modalities such as text, images, video, and audio, this field is known as Multi-Modal Information Retrieval (MMIR). Research towards MMIR methods is gaining considerable interest due to the expansion of data, resulting in a need for efficient methods capable of handling large-scale multi-modal data [1,2,3,4,5]. Cross-Modal Retrieval (CMR) is a sub-field of MMIR which focuses on retrieving information from one modality using a query from another modality. An example of CMR is retrieving images when using text as a query and vice versa. These image-to-text and text-to-image tasks are the focus of CMR within this paper.

Where retrieval speed and storage space are considered top priorities, Cross-Modal Hashing (CMH) networks have recently been favoured over other traditional retrieval methods due to the computational efficiency and compactness that comes with the binary representations produced by hash-based methods [6,7]. CMH networks are typically constructed with parallel text and image modules that work in unison to learn the objective hash function. Through the use of the hash function, image and text samples can be mapped to a joint hash subspace [8]. Within this subspace, similarity comparisons can be made when prompted by a query to rank data points by relevance for the top results to be provided as the result of the retrieval task.

Recent state-of-the-art CMH methods include Deep Cross-Modal Hashing (DCMH) [9], Adversary Guided Asymmetric Hashing (AGAH) [10], Joint-modal Distribution based Similarity Hashing (JDSH) [11] and Deep Adversarial Discrete Hashing (DADH) [12]. Jiang et al. [9] proposed DCMH by applying a deep learning approach to feature learning and hash generation, an end-to-end deep learning framework for CMH. As opposed to previous CMH methods that employed a relaxation of the discrete learning problem into a continuous learning problem, hash code generation within DCMH was learnt directly without relaxation. DCMH laid the foundation for forthcoming methods such as AGAH proposed by Gu et al. [10], which through an adversarial approach to learning and the introduction of three collaborative loss functions, similarities of similar pairs were strengthened while disassociating dissimilar pairs. Liu et al. [11], with the proposed JDSH network, employed a joint-modal similarity matrix to preserve semantic correlations and exploit latent intrinsic modality characteristics. Further, the Distribution-based Similarity Decision and Weighting (DSDW) module was proposed as part of JDSH to generate more discriminative hash codes. Bai et al. [12], with the adversarial-based method DADH, maintained semantic consistency between original and generated representations while making the generated hash codes discriminative.

The data most often used during training of information retrieval networks are paired, e.g., there is one-to-one or one-to-many correspondence between the text and image samples being used. However, such paired data are not always present in real-world data, and often constructed for training machine learning models. Unpaired data where no relationship is given between text and image samples are a common scenario present in the real world, which is not currently accounted for in many proposed CMH methods. Once unpaired samples are introduced into the data being used, pairwise-constrained methods cannot process such unpaired data in their baseline configuration and could therefore be unsuitable for real-world use cases where the data to be used are unpaired.

This paper proposes a framework that facilitates the use of unpaired data samples for the training of CMH methods. The proposed framework can be employed to enable CMH methods to include unpaired samples in the learning process. The contributions of this paper are as follows.

1. A comprehensive overview of CMH methods, specifically in the context of utilising unpaired data. The current state of CMH is surveyed, the different pairwise relationship forms in which data can be represented are identified, and the current use or lack of unpaired data is discussed [6,13,14]. However, the literature does not provide an overview of CMH methods applied to unpaired data. The aspects which bind current CMH methods to paired data are discussed.
2. A new framework for Unpaired Multi-Modal Learning (UMML) to enable training of otherwise pairwise-constrained CMH methods on unpaired data. Pairwise-constrained CMH methods cannot inherently include unpaired samples in their learning process. Using the proposed framework, the MIR-Flickr25K and NUS-WIDE datasets are adapted to enable training of pairwise-constrained CMH methods when datasets contain unpaired images, unpaired text, and both unpaired image and text within their training set.
3. Experiments were carried out to (1) evaluate state-of-the-art CMH methods using the proposed UMML framework when using paired and unpaired data samples for training, and (2) provide an insight as to whether unpaired data samples can be utilised during the training process to reflect real-world use cases where paired data may not be available but a network needs to be trained for a CMR task.

This paper is organised as follows: Section 2 surveys the current state of CMH methods and unpaired data usage. Section 3 provides an overview of the proposed UMML framework. Section 4 discusses the datasets, the experiment methodology and the evaluation metrics used. Section 5 describes experiments that have been conducted using state-of-the-art CMH methods employing the proposed UMML framework for training across various data pairing and unpairing scenarios.

## 2. Related Work

The critical step to conducting the task of CMH retrieval is to establish and learn a common latent subspace onto which features from different modalities can be mapped [15,16]. This common latent subspace is traditionally constructed by learning to correlate the binary representation of samples from different modalities as explored in the method Canonical Correlation Analysis (CCA) [17], where similarity comparisons can be made within the latent subspace to perform the task of retrieval. Although robust in performance, Deep Learning Information Retrieval methods have been criticised for their computational complexity and the difficulty of scaling these to large-scale datasets [18]. As a counterpart to this, hashing techniques are often favoured for their low storage cost and fast query speeds. The following are two different vital approaches which encompass search through hashing [19]:

**Hamming distance sorting.** When comparing two binary string hash codes, hamming distance refers to the number of bits that are different between the two codes, where a short hamming distance represents similarity and a long hamming distance represents divergence. Hamming distance sorting can be used as the nearest neighbour approach, where the hamming distances of the query sample relative to the samples in the retrieval dataset are obtained and ranked. The top samples in the formed rank would be the samples to be retrieved. Hamming distance sorting is computationally efficient and therefore meets the aim of hashing-based retrieval, that being fast query speeds.

**Hash table retrieval.** Hash table retrieval aims to obtain a reduction in the number of distance computations needed when performing the retrieval task. This reduction is achieved through hash tables which sort samples into buckets according to their similarities. Samples closest in similarity will be placed in the same bucket, whereas divergent samples will be placed in different buckets. When a query is made, the most similar bucket that is relative to the query will be selected as the retrieval candidate. By only performing similarity computations among the hash tables and the hash table candidate contents, computing the similarity of the rest of the dataset is avoided.

### 2.1. Multi-Modal Pairwise Relationship Types

**Figure 1:**
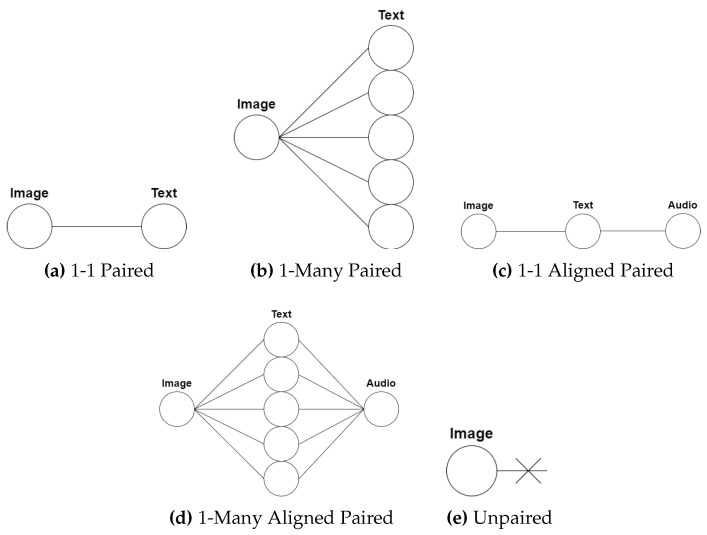
The various pairwise relationships present in information retrieval datasets. (**a**) 1-1 Paired, (**b**) 1-1 Many Paired, (**c**) 1-1 Aligned Paired, (**d**) 1-Many Aligned Paired, and (**e**) Unpaired.

This section presents a classification of multi-modal data in terms of pairwise relationships. The discussion will focus on information retrieval, however the provided classification can be applied to other multi-modal applications. The pairwise relationship of the samples in a dataset dictates the approach to be employed when training a network for an information retrieval task. If pairwise relationships exist, these can be leveraged for improved learning of the task at hand. If pairwise relationships do not exist, i.e., the dataset is unpaired, the approach to designing multi-modal methods for such data will be different. Therefore, it is important to identify the relationship type of the data in use to address the task appropriately. The pairwise relationships are illustrated in Figure 1 and described as follows.

**1–1 Paired Samples.** This describes a relationship when a sample from one modality (e.g., an image) has its direct counterpart in another modality (e.g., text describing the image) as seen in Figure 1a. When measuring similarity, the paired sample’s similarity score is the highest compared to the rest of the samples. Using paired samples during training of CMH methods allows for leveraging the paired relationship when mapping samples from the input space to the hashed subspace. By leveraging this paired relationship, samples that are similar in the input space will also remain similar in the hashed subspace, allowing for similarity comparisons in the hashed subspace. An example of a pairwise relationship dataset is the Wiki dataset, which is made up of image and text pairs from Wikipedia article cover pictures and article texts [20].

**1–Many Paired Samples.** This refers to a relationship where a sample (e.g., an image) has more than one counterpart in another modality (e.g., multiple text descriptions relevant to the particular image) as shown in Figure 1b. The cross-modal similarity scores between a sample and its many paired counterparts are the highest compared to the rest of the samples. An example of a one-to-many pairwise relationship dataset is the Flickr30K dataset [21], that is made up of image and five caption paired samples.

**1–1 Aligned Paired Samples.** In the case of having more than two modalities, this relationship refers to samples with an aligned one-to-one pairwise basis across the different modalities in the dataset. For example Figure 1c, the image is paired to the text, and the text is paired to the audio, and therefore it follows that the image is paired to the audio. An example of such a dataset is the PKU XMediaNet dataset [22], which is made up of paired aligned image, text, audio, video and 3D model samples.

**1–Many Aligned Paired Samples.** In the case of having more than two modalities, this relationship refers to samples with an aligned one-to-many pairwise basis across the different modalities present in the dataset. When considering Figure 1d, the image has a one-to-many pairing with the text, the text has a many-to-one pairing with the audio, and thus it follows that the image is paired to the audio. The literature mainly discusses the use and evaluation of datasets that contain 1–1, 1–many and 1–1 aligned pairwise relationships, while 1–many aligned paired relationships are seldom discussed [19,23].

**Unpaired Samples.** These are fully unpaired samples that are independent in their respective modality and have no direct counterpart in another modality as shown in Figure 1e. Therefore, using these samples to learn cross-modal transformations results in a difficult task to approach when compared to the paired scenario due to the paired relationship not being leveraged to maintain inter-modal similarity.

### 2.2. Learning to Hash

Learning to hash refers to learning a hash function y=h(x) through which input item *x* is mapped to hash code *y*. As discussed by Wang et al. [6], the following are the primary considerations when approaching the task of learning to hash: the hash function to be used, the similarity adopted for both the input and the hash space, the loss function to adopt the optimisation objective, and the optimisation technique employed.

**Hash function.** The hash function aims to generate hash codes such that nearest neighbour samples in the input space are the closest to each other within the hash subspace. Hashing-based methods have traditionally employed linear projection, kernels, spherical functions and a non-parametric function for their hashing functions [6,24,25]. However, recent research has steered towards the use of deep neural networks as hash functions [23] for two main reasons: firstly, the ability to learn very complex hash functions through the strong representation capabilities of deep learning-based methods; and secondly, the possibility of employing end-to-end hash code generation frameworks [9].

**Similarity measures.** Similarity measures for the input space and the hash coding space need to be considered. Similarity functions for hashing are most commonly based on the distance between two given samples, which often comes from Euclidean distance. The similarity functions frequently used include the Gaussian function and cosine similarity. The similarity value for deep neural network-based hashing is usually binary, with 1 representing common semantic meaning and 0 otherwise. For the hash code space, hamming distance is commonly used: Let $y_{i}$ be a hashed image sample and $y_{j}$ be a hashed text sample. The Hamming distance $d_{ij}^{h}$ between $y_{i}$ and $y_{j}$, is calculated as:

(1) $$d_{ij}^{h}=\sum_{m=1}^{M}\delta \left[y_{im}\neq y_{jm}\right]$$

where *M* is the bit length of the hash codes to be compared and $\delta [y_{im}\neq y_{jm}]$ is the case of having different bit values. Hamming distance provides the number of different bit values when comparing two hash codes, and similarity based on hamming distance is defined as $s_{ij}^{h}=M-d_{ij}^{h}$, that is, the number of equal bits.

**Loss function.** The main aim when employing the loss function for hashing purposes is to maintain the similarity of samples within the original input space when mapped to the latent subspace, i.e., the nearest neighbours in the subspace should also be the nearest neighbours in the original input space. A widely adopted technique for maintaining input-subspace similarity is pairwise similarity preserving [6], which aims to make the distances or similarities between samples in their original input and hash representations as consistent as possible. Pairwise similarity preserving consists of assigning a small hamming distance to similar images with similar hash codes, and assigning a larger hamming distance to divergent images with contrasting hash codes.

**Optimisation.** There are two main issues to be considered with deep hashing methods which call for optimisation. The first issue is the vanishing gradient problem (gradients becoming vanishingly small during the training process, preventing any further learning) stemming from the sign function employed for learning to hash. The second issue is the high time complexity when dealing with large amounts of data [6]. A common approach to solving the vanishing gradient problem is continuous relaxation [26], which comes in the form of sigmoid relaxation, tanh relaxation, and directly dropping the sign function sgn(*z*) ≈*z*.

### 2.3. Cross-Modal Hashing Categorisation

Following the basis set by Cao et al. [19] in their 2020 survey on hashing methods for multi-modal retrieval, hashing approaches can be categorised into data-independent, data-dependent and deep hashing methods.

Data-independent methods tackle the task of learning to hash independently of the data in use, where the hash function is generally generated through random mapping. The characteristic approach to such a method is using Locality-Sensitive Hashing (LSH) [27]. Locality-Sensitive Hashing is based on assigning samples with high similarity to the same hash bucket in hash table retrieval, which ensures neighbouring samples in the original space remain as close as possible to each other in the hash space. Although this makes locality-sensitive hashing reliable, the issue of hash collision arises when large data sets are used, where longer hash codes need to be used to avoid collision. The need for longer hash codes results in additional computational resources being required, which results in a decrease in performance.

Data-dependent methods make use of the information available in the dataset as training data to generate the hash function to be used, as opposed to data-independent methods which cannot leverage the information in the dataset for hash function generation. Being able to use this information generally results in improved performance. The following are some characteristic approaches to data-dependent methods: with Cross View Hashing (CVH) [4] the hash function is learnt by minimising the weighted average hamming distance between the different modalities. Linear Cross-Modal Hashing [28] first partitions the training data into clusters to calculate the distance between the data point being processed and the centroids of the formed clusters to maintain the similarity inside modalities. Collective Matrix Factorisation Hashing (CMFH) [29] is an unsupervised method that assumes the hash codes for modalities are consistent, and the collective matrix factorisation method is used to construct the hash function model.

**Figure 2:**
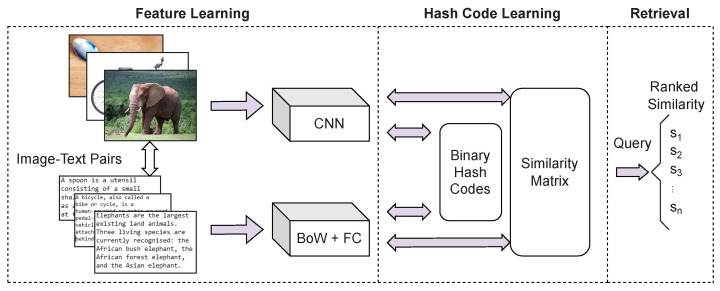
Overview of an end-to-end deep hashing architecture. This figure illustrates a simplified recreation of the Deep Cross-Modal Hashing (DCMH) [9] network architecture (CNN: Convolutional Neural Network, BOW: bag of words, FC: Fully Connected layers). Example elephant (1), bicycle (2) and spoon (3) images reprinted under Creative Commons attribution, (1) Title: Elephant Addo, Author: Mikefairbanks, Source, CC BY 2.0 (2) Title: Dessert Spoon, Author: Donovan Govan, Source, CC BY-SA 3.0 (3) Title: Electric Bicycle, Author: Mikefairbanks, Source, CC BY-SA 3.0.

Deep hashing methods came as a result of the recent success of deep neural networks across a multitude of fields and are currently the category of cross-modal hashing methods which is receiving attention [30,31,32,33,34]. Deep features extracted by deep hashing methods contain abundant semantic information and can more accurately represent the original data when compared to traditional linearly derived hash functions. Therefore, the use of deep learning within hashing methods can result in considerable retrieval performance improvement. The following are some of the characteristic approaches to deep hashing methods: Deep Cross-Modal Hashing (DCMH) [9] integrates feature learning and hash learning into an end-to-end deep learning framework with the use of text and image modality data as shown in Figure 2. The end-to-end nature of this approach means the different parts of the model can work in unison, providing feedback to each other during the learning process. This approach has been adopted in principle by many subsequently published methods. Pairwise Relationship Deep Hashing (PRDH) [35] came as an improvement to DCMH, that can integrate different types of pairwise constraints to better reflect the hash code similarity from inter- and intra-modal data. Self-Supervised Adversarial Hashing (SSAH) [36] uses mechanisms such as self-supervised semantic generation and adversarial learning, which resulted in significant advances in retrieval performance.

### 2.4. Unpaired Cross-Modal Hashing Methods

Generalized Semantic Preserving Hashing (GSPH) proposed by Mandal et al. [37] identified two unpaired sample scenarios, namely Single label-Unpaired (SL-U) and Multi Label-Unpaired (ML-U), and was the first method to address both cases. GSPH supports training with unpaired samples; however, an extra unification step is also proposed to improve performance if paired data are present. To explore the unpaired scenario, samples from one modality were dropped while samples from the counterpart modality were kept.

Hu et al. [38] with their proposed method Triplet Fusion Network Hashing (TFNH), explore the possibility of conditioning the retrieval space of the datasets in use for these to include a certain percentage of unpaired samples. The performance of their method was evaluated for the task of retrieving unpaired samples through a multi-step evaluation which discarded 10% of paired relationships in each step. Compared to using fully paired data, a gradual performance drop was observed in TFNH when unpaired data were utilised to train the model.

Wen et al. [39], with their proposed method Cross-Modal Similarity Transferring (CMST), take a transfer learning approach towards unpaired cross-modal retrieval to aid the training process. Using intra- and inter-modal similarity learning networks, knowledge obtained from unpaired samples improved retrieval performance across the board over the different datasets and setting scenarios. The most noteworthy improvement was made on the Pascal Sentences dataset, with an average improvement of 12% on the image-text retrieval task. These improvements were another indication that developing retrieval methods that can use unpaired samples would be of significant use.

Gao et al. [40] proposed the method Unpaired Cross-Modal Hashing (UCMH), which tackles the issue of unpaired samples by employing a multi-step process. First, the latent subspaces for the different modalities are learnt separately. The latent subspaces are learnt through similarity preservation [41], that is used to preserve the intra-modality similarity. Matrix factorisation is then used to improve the discriminative ability of the learned hash codes. Finally, an affinity matrix is constructed to bridge the modality gap.

Robust Unsupervised Cross-modal Hashing (RUCMH) proposed by Cheng et al. [42] introduces an unsupervised two-part approach to learning. Firstly, modality-specific hashing functions are constructed through a transfer learning-based approach. Secondly, objects from one modality are linearly represented through samples of the counterpart modality in the shared subspace. Percentages of training data were shuffled in increments of 10%. While other CMH methods saw a decrease in performance relative to the amount of shuffling in the training set, RUCMH was unaffected by the shuffling as it is an unsupervised method and is independent of pairwise relationships.

Liu et al. [18] propose the framework Matrix Tri-Factorization Hashing (MTFH), which encompasses the different pairwise relationship scenarios, including the unpaired scenario, using matrix tri-factorisation hashing. The focus of this method is the use of varying-length hashing, a previously relatively unexplored approach in an area that favoured equal-length hashing. The experiments by Liu et al. showcased the importance of finding the optimal combination of image/text hash code lengths hash codes that are either too short or too long could result in degrading performance.

Adaptive Marginalized Semantic Hashing (AMSH) proposed by Luo et al. [43] introduced multiple regression models to learn modality-specific representations. Most previous methods constructed a linear regression between the latent feature space and the label space to learn a common latent representation, an approach that is not feasible on the unpaired data scenario. When exploring the unpaired scenario, datasets were randomly shuffled to sever 50% of the one-to-one data correspondences.

Flexible Cross-Modal Hashing (FlexCMH) proposed by Yu et al. [44] leveraged centroids of clusters through a matching strategy for training. Unlike previous clustering-based methods, training of FlexCMH could be conducted regardless of whether the number of samples in the constructed clusters or modalities was equal, a constraint that was present in previous clustering-based methods. FlexCMH can flexibly adapt to the number of pairs present to maximise learning. FlexCMH does this by unpairing data through shuffling the data to remove the pairwise relationships and removing a portion of the text modality.

### 2.5. Architectural Reliance on Paired Samples of Existing CMH Methods

This section describes the learning process of three CMH methods for information retrieval and the aspects which bind these methods to the use of paired samples. The methods Deep Adversarial Discrete Hashing (DADH) [12], Adversary Guided Asymmetric Hashing (AGAH) [10] and Joint-modal Distribution based Similarity Hashing (JDSH) [11] were chosen due to their relevance within the CMH information retrieval field, and due to the full source code for each of the methods being publicly available.

**Figure 3:**
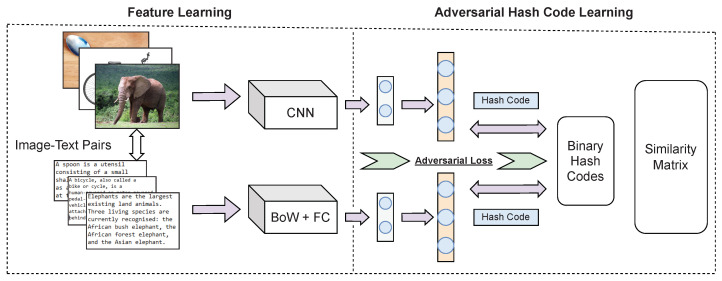
Simplified workflow of adversarial-based CMH methods depicting approaches used by methods such as Deep Adversarial Discrete Hashing (DADH) [12] and Adversary Guided Asymmetric Hashing (AGAH) [10] (CNN: Convolutional Neural Network, BOW: bag of words, FC: Fully Connected layers).

**DADH [12].** The typical workflow of adversarial-based CMH methods is illustrated in Figure 3, which DADH follows. The architecture comprises a feature-learning module and a Hashing-learning module. For the feature-learning module, the original input of image features or text features was processed into low-dimensional features by a convolutional neural network. Several loss functions were used for the hashing-learning module to generate compact binary codes with reduced quantisation loss. To ensure cross-modal consistency, adversarial learning is introduced in feature- and hashing-learning modules. This use of adversarial learning differs from other methods, which only use adversarial learning in the feature learning phase. The adversarial step is conducted as follows: given a feature projection of a sample from one modality, which is the true feature, the feature projection from its paired counterpart modality is used as the fake feature to discriminate against. The adversarial layer then attempts to identify the origin modality of the sample being processed. If the origin modality is unclear, the gap between the image and text modalities is considered to have been appropriately bridged.

**AGAH [10].** Similarly to DADH, AGAH follows the adversarial-based CMH workflow as illustrated in Figure 3. The feature extractor comprises two deep neural networks, the image modality network and the text modality network. An adversary-guided attention module joins these two networks. This way, the feature learning procedure is enhanced, ensuring both the cross-modal consistency and the maintenance of semantic distinctiveness of extracted features. A convolutional neural network (CNN) is adopted for image feature learning, and a multi-layer perceptron model was proposed for the text modality with three fully-connected layers to learn the text feature representations. To generate modality-consistent representations across different modalities, two classifiers were designed, one for each modality, based on an adversarial approach similar to that used within DADH. For the image modality classifier, the text network was used as the image features generator. The extracted features from the image network are considered real, while the extracted features from the text network are marked as fake. The image modality classifier aims to distinguish whether the input image features are real or fake. The text modality classifier behaves similarly to the image modality classifier. To ensure the semantic relevance preservation between each feature representation and its label information, an attention module guided by the adversarial was designed to aid the learning process. The attention module assigns each item of the representation vector a weight according to the distance between the representations.

**JDSH [11].** The main contributions of JDSH include the construction of a joint-modal similarity matrix used for supervised hash code generation and the proposition of the Distribution-based Similarity Decision Weighting (DSDW) method used for strengthening the generated hash codes. The joint-modal similarity construction process starts with separate text and image cosine similarity matrices derived from pre-trained deep neural network feature extraction. The image modality similarity matrix $S_{v}$ and text modality similarity matrix $S_{t}$ are then fused into the final joint-modal similarity matrix S defined as:

(2) $$\begin{array}{cc} & S=\alpha S_{v}+\beta S_{t}+\gamma S_{\mathrm{fusion}\;}={\left\{s_{ij}\right\}}_{i,j=1}^{N} \end{array}$$

(3) $$\begin{array}{cc} & \alpha ,\beta ,\gamma \geq 0,\alpha +\beta +\gamma =1,s_{ij}\in \left[-1,1\right] \end{array}$$

where $s_{ij}$ is the image-text pair similarity of image *i* and text *j*, *N* is the total amount of data pairs, α, β, γ are constant parameters for similarity importance control between the different modalities, and $S_{fusion}$ is a symmetric matrix. The symmetric matrix is constructed on the basis that two instances in one modality should obtain similar similarity relations in their counterpart modality. The joint-modal similarity matrix $S={\left\{s_{ij}\right\}}_{i,j=1}^{N}$ is the foundation for the driver of hash code generation framework DSDW.

The methods DADH and AGAH at their core are adversarial networks. The adversarial step in both methods is based on using an image and text pair to construct a true feature originating from the modality to be processed and a fake feature obtained from the counterpart modality for learning. Therefore, if the sample to be processed is unpaired, the true and fake feature pair cannot be formed, thus introducing a pairwise constraint at the adversarial step. In the case of JDSH, the pairwise constraint resides in the formulation of the joint-modal similarity matrix $S={\left\{s_{ij}\right\}}_{i,j=1}^{N}$ as seen by the use of the image-text pair similarity $s_{ij}$, which is the driver of the hash code generation framework used.

## 3. UMML: Proposed Unpaired Multi-Modal Learning (UMML) Framework

**Figure 4:**
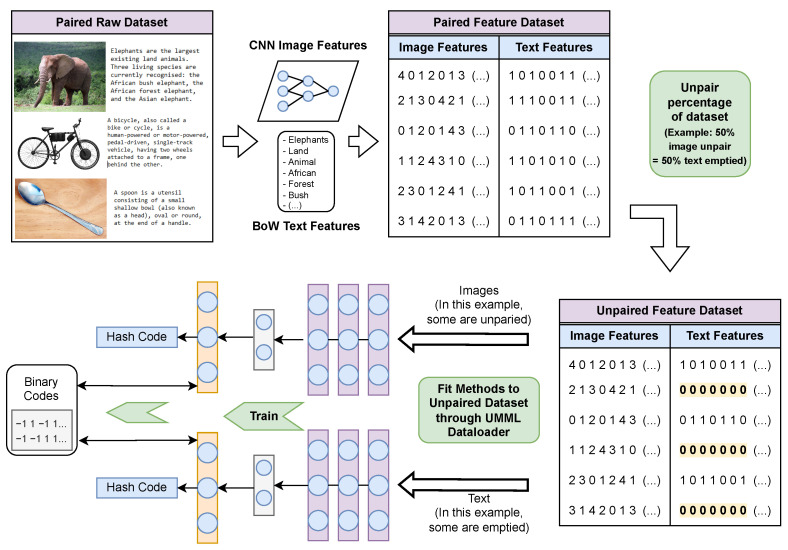
Unpaired Multi-Modal Learning (UMML) framework workflow. The diagram shows an example of 50% of images being unpaired where 50% text Bag of Words (BoW) binary vectors are emptied. Similarly, in the case of text being unpaired, the image feature matrices would be emptied (CNN: convolutional neural network).

To make CMH methods compatible with the processing of unpaired samples, a suitable approach must be employed to include unpaired samples in the datasets to be used. The need for a suitable approach is due to CMH methods often not being compatible with the processing of unpaired samples, as is the case with methods DADH, AGAH and JDSH as discussed in Section 2.5. The input to the methods tested are pairs of image and text, and as such, the methods do not allow for a single unpaired sample to be used for training. To tackle this issue, the Unpaired Multi-Modal Learning (UMML) framework is proposed, illustrated in Figure 4. The implementation of this framework is provided (https://github.com/MikelWL/UMML, accessed on 9 December 2022).

**The unpairing process.** Let *X* be a n×i matrix where *n* is the number of image samples and *i* is the number of image features, and let *Y* be a n×j matrix of *n* text samples and *j* text vectors. The dataset *D* to be unpaired is defined as D=(X,Y), where each row $X_{n}\in X$ is paired to each row $Y_{n}\in Y$. The samples to be unpaired are selected according to the percentage of the training set being unpaired: if 20% of data is unpaired, the first 20 out of every 100 samples of the training set are selected to be unpaired, and if 40% of data is unpaired, the first 40 out of every 100 samples are selected, and so on. Once the samples to be unpaired are selected, in the case of $X_{n}$ images being unpaired, text vectors are extended to include unpaired sample markers at the end of the vector, and the corresponding paired $Y_{n}$ text vectors are replaced by an empty vector and marked as unpaired samples. In the case of $Y_{n}$ texts being unpaired, the corresponding paired $X_{n}$ image features are replaced by an empty vector and marked as unpaired. Finally, the dataset is constructed with the newly unpaired samples, and appropriate data loaders provided within UMML are used to feed the unpaired datasets to the methods being trained. These data loaders ensure the input requirements of the methods match the dimensions of the newly constructed unpaired dataset.

UMML is needed because pairwise-constrained CMH methods do not support actual unpaired data, thus requiring the use of empty samples to enable unpaired scenario evaluations. Once these unpaired samples are fed to the method being trained, no semantic value will be present within the emptied samples, resulting in semantically unpaired training. The newly unpaired samples are finally bundled with the samples which have been kept paired, and the unpaired dataset variant is constructed. Note that only the training set of the dataset is made unpaired by the UMML framework, as the query/retrieval set is left unaltered to avoid using empty samples as queries/retrievals during testing.

## 4. Experiment Methodology

### 4.1. Datasets

MIRFlickr-25K [45] contains 25k image-tag pairs, with these samples being assigned to at least one label from 24 classes. ‘Tags’ refer to the tags associated with each image, which are used as the text counterpart to the images for retrieval. ‘Labels’ are used as ground truth (i.e., classes) to measure performance at the test stages. For the experiments conducted, only the image-tag pairs which contain at least 20 textual tags are used, which brings down the overall image-tag pair count to 20,015. The query set consists of the samples which are used as the queries for the test stage, and the retrieval set contains the retrieval candidates to the query. The training set is formed as a subset of the retrieval set.

NUS-WIDE [46] consists of 269,648 image-tag pairs categorised into 81 manually annotated classes. For our experiments, 195,834 image-tag pairs are selected, which belong to the 21 most frequent concepts. Table 1 shows the number of samples utilised for training and testing of the MIR-Flickr25K and NUS-Wide datasets. Table 2 shows examples of MIR-Flickr25K and NUS-Wide images, along with their tags and labels.

### 4.2. Methods

The experiments compare state-of-the-art cross-modal hashing methods for information retrieval and evaluate their performance in paired and unpaired data scenarios using the proposed UMML framework. These methods are: Adversary Guided Asymmetric Hashing (AGAH) [10], Joint-modal Distribution-based Similarity Hashing (JDSH) [11] and Deep Adversarial Discrete Hashing (DADH) [12]. These methods were chosen due to their relevance within the CMH information retrieval field and the full source code for each of the methods being publicly available. Unsupervised methods such as JDSH could opt to drop samples, i.e., skip samples in the training process, to replicate unpaired sample behaviour. During experiments using the proposed UMML framework, samples were not dropped to ensure consistency when comparing the methods. For all the methods evaluated, the 64-Bit setting is used. Experiments were not conducted with other bit settings because the study focuses on the usage and effect of training with unpaired samples instead of comparing the efficiency of different bit lengths.

### 4.3. Evaluation Metrics

To evaluate the performance of the CMH models, the widely adopted [12] retrieval procedure of Hamming ranking is used, that sorts search results based on Hamming distance to the query samples. The metric mean average precision (mAP) is then used to evaluate Hamming retrieval performance. Precision is the fraction of retrieved samples that are relevant to a query. Recall is the fraction of the relevant samples that are successfully retrieved. Average Precision (AP) is the mean of the precision scores for a given query. Mean Average Precision(mAP) is the mean of the AP across a number of queries. Mean average precision is the primary performance measure employed in CMH retrieval, and it is obtained as the mean of the average precision (AP) values at 100% Recall.

Performance difference compares the results when training with unpaired samples to results when training with paired samples. Let $mAP_{p}$ be the performance obtained during paired training and $mAP_{u}$ be the performance obtained during unpaired training. The percentage of performance difference is calculated as follows.

(4) $$\mathrm{Perf}.\mathrm{Diff}.=\left(\frac{mAP_{u}}{mAP_{p}}-1\right)\times 100$$

## 5. Experiment Results

This section presents the results of experiments conducted using the proposed UMML framework applied to facilitate unpaired learning using the DADH, AGAH and JDSH methods and the MIR-Flicker25K and NUS-WIDE datasets. The training performance of these CMH methods is evaluated across different sampling scenarios: unpaired images, unpaired text, a combination of unpaired images and text, and random sample discarding. The abovementioned methods are also compared to other unpaired CMH methods that can learn unpaired data and thus do not require the UMML framework. Finally, the performance of the most promising method, DADH, is investigated at a more granular level that involves analysing DADH’s performance across each class of the MIR-Flickr25K dataset.

### 5.1. Training with Unpaired Images

**Figure 5:**
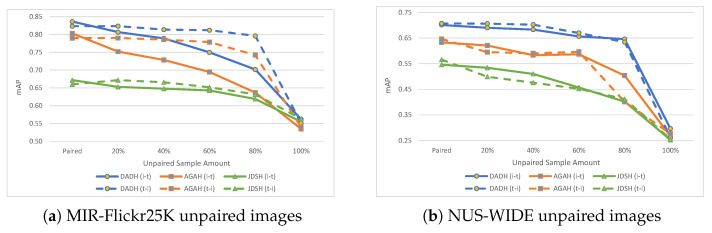
Results (mAP) on MIR-Flickr25K and NUS-WIDE with unpaired images, i.e., images with no corresponding text. The ‘Paired’ points show results when training with a fully paired training set. Subsequent points show results with increasing amounts of unpaired images in the training set in increments of 20%.

Image to text (i→t) and text to image (t→i) evaluation results using unpaired images within the training set are presented in Table 3 and illustrated in Figure 5 for the MIR-Flickr25K and NUS-WIDE datasets. On the left-hand side of Table 3, results when training with a fully paired training set are provided. Results when using different unpaired sample sizes are then shown in increments of 20%. DADH, being the method built as an improvement to AGAH, outperforms the other methods, followed by AGAH. JDSH sees more limited performance when compared to DADH and AGAH, mainly due to it being an unsupervised method.

For MIR-Flickr25K, as shown in Figure 5a, DADH and AGAH show similar behaviours when using unpaired images for training; (t→i) results see a marginal decrease in performance, while the (i→t) task sees a more gradual decrease in performance as more unpaired images are introduced to the training set. In the case of JDSH however, both tasks are affected in a similar manner. For NUS-WIDE, the results obtained show a different pattern when compared to that observed in MIR-Flickr25K. For DADH, AGAH and JDSH, the performance decrease as more unpaired images are introduced is gradual for both (i→t) and (t→i) tasks. Based on the results shown in Table 3 and Figure 5, the following main observations can be made when training with unpaired images.

(1) Dataset impacts the performance of models. Different datasets provide different behaviours when unpaired samples are introduced into the training set. With MIR-Flickr25K, DADH and AGAH see different patterns of performance decrease for the (i→t) and (t→i) tasks, while with NUS-WIDE, DADH and AGAH see similar patterns for the two tasks. JDSH, on the other hand, shows similar patterns for both tasks on both datasets.
(2) Percentage of Unpairing may impact performance. For MIR-Flickr25K, the performance of methods DADH and AGAH for the (i→t) task is negatively affected as the percentage of unpaired images increases. For the (t→i) however, with the exception of 100% image unpairing, performance was unaffected when the percentage of unpaired images increased. Once all images in the training set are fully unpaired (i.e., 100% unpaired), the performance of both tasks across all methods is measured at an average of 0.564 mAP for MIR-Flickr25K and 0.268 mAP for NUS-WIDE. These results will later be compared to random performance evaluations in Section 5.4 to determine the extent to which the methods are learning from training with 100% unpaired images.

### 5.2. Training with Unpaired Text

**Figure 6:**
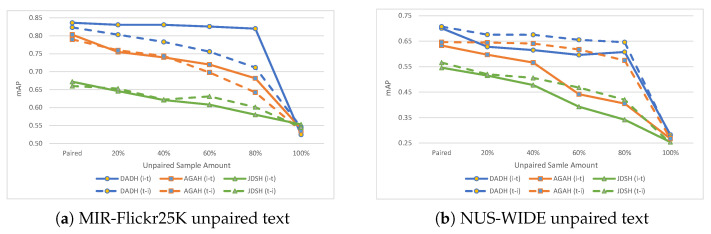
Results (mAP) on MIR-Flickr25K and NUS-WIDE with unpaired text, i.e., text with no corresponding images. The ‘Paired’ points show results when training with a fully paired training set. Subsequent points show results with increasing amounts of unpaired text in the training set in increments of 20%.

Evaluation results using unpaired text within the training set are presented in Table 4 and shown in Figure 6. For MIR-Flickr25K, in the case of DADH, (i→t) retains its performance as more unpaired samples are used for training while the (t→i) is the task which sees a gradual performance decrease. This behaviour is the opposite of what occurred when unpaired images were used for training, which indicates that the task mostly affected by using unpaired samples for training in the case of DADH is the task which uses the unpaired modality as the query of the retrieval. In the case of AGAH and JDSH, however, both tasks saw similar rates of performance decrease as more unpaired samples were used for training. This behaviour deviates from what would be expected when considering the unpaired text scenario, where AGAH saw the two tasks being affected differently. For NUS-WIDE, on the other hand, for all three methods DADH, AGAH and JDSH, both (i→t) and (t→i) tasks saw a performance decrease with the (i→t) task being the task which saw the most decrease.

In addition to the observations made when unpairing images, unpairing text provides the following observation: whether (i→t) or (t→i) will be the most affected task when training with unpaired samples depends on the method used, the dataset being evaluated, and the modality being used. In the case of DADH, when training with unpaired text on the MIR-Flickr25K dataset, it was the (t→i) task that was most negatively affected. For AGAH, however, when unpairing text on the NUS-WIDE dataset, it was the (t→i) task that was most negatively affected. It is essential to evaluate the method being studied to verify its behaviour when training with unpaired samples. This behaviour will determine whether it is feasible to adapt the CMH method to the unpaired sampling scenario.

### 5.3. Training with Unpaired Images and Text

**Figure 7:**
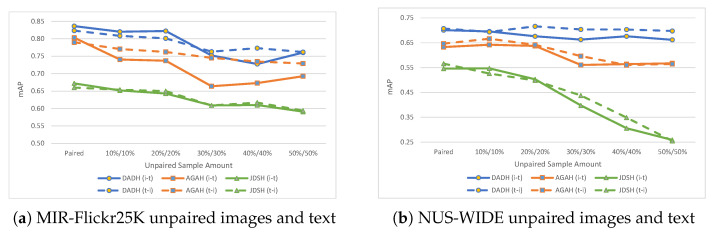
Results (mAP) on MIR-Flickr25K and NUS-WIDE with unpaired images and text, i.e., images with no corresponding text and vice versa. The ‘Paired’ points show results when training with a fully paired training set. Subsequent points show results with increasing amounts of unpaired images and text in the training set, for example, ‘10%/10%’ refers to 10% of the training set being unpaired images and another 10% being unpaired text for a total of 20% of the dataset being unpaired samples.

Evaluation results using unpaired images and text within the training set are presented in Table 5 and shown in Figure 7. A noteworthy observation is concerning the 50%/50% training set that comprises 50% of unpaired images and 50% unpaired text. DADH and AGAH do not see the same drop in performance as was seen with the 100% image or 100% text unpaired evaluations (see Table 3), where performance dropped to an average of 0.546 mAP for MIR-Flickr25K and 0.267 mAP for NUS-WIDE. Instead, performance when using the 50%/50% training set was measured at an average of 0.761 mAP for MIR-Flickr25K and 0.680 for NUS-WIDE with DADH, and 0.711 mAP for MIR-Flickr25K and 0.566 for NUS-WIDE using AGAH. These results indicate that DADH and AGAH can learn more from the 50%/50% unpaired training set when compared to the 100% unpaired image and 100% unpaired text sets.

### 5.4. Training with Sample Discarding

Previous experiments evaluated the retrieval performance of CMH methods when using unpaired samples during the training process. Overall, a gradual decrease in performance was observed when the percentage of unpaired samples increased within the training set. The objective of the experiment described in this section is to investigate whether including unpaired samples in the training set improves retrieval performance compared to discarding them.

**Figure 8:**
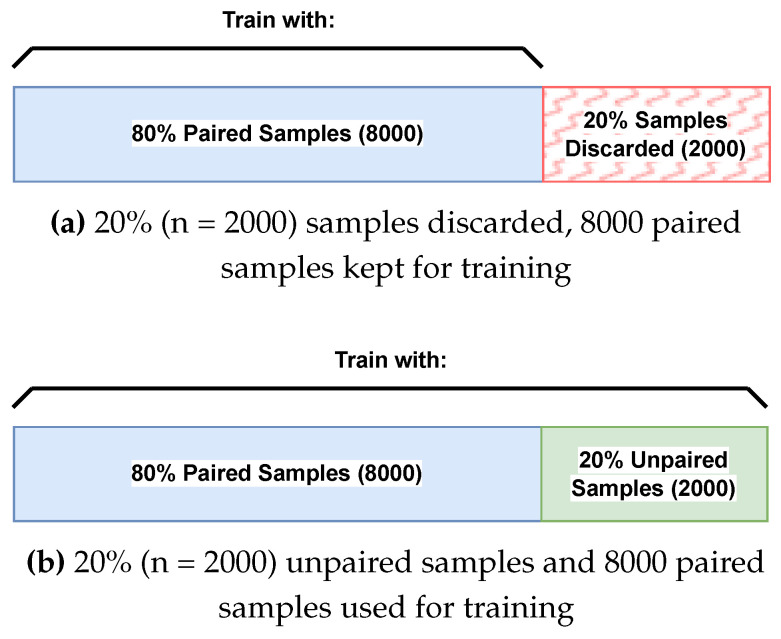
In (**a**), 20% of the training set was discarded. In (**b**), 20% of the training set was unpaired. In this example, for both (**a**,**b**), the model will be trained on 8000 paired samples. However, (**b**) will also train with its additional 2000 unpaired samples. This way, the effect of training with or without the additional unpaired samples can be investigated.

**Method for Sample Discarding.** Sample discarding refers to removing a given percentage of paired samples from the training set, which results in a smaller but still fully paired training set. For example, the datasets utilised for the experiments comprise 10,000 paired samples each. When 20% (2000) of samples are discarded, the remaining 8000 pairs will be utilised for training. Training when discarding 20% of pairs can then be compared to training with a set of which 20% of samples are unpaired. This comparison is illustrated in Figure 8. The random performance baseline was created by performing cross-modal retrieval using the test set on each model before training.

**Figure 9:**
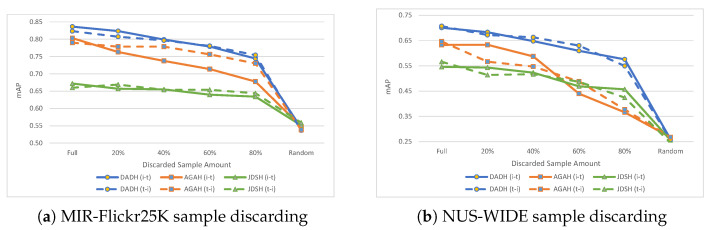
Results (mAP) on MIR-Flickr25K and NUS-WIDE with sample discarding, i.e., training set being reduced. The ‘Full’ points show results when training with the full unaltered training set. Subsequent points show results with decreasing amounts of samples, where the given percentage denotes the percentage of samples in the training set which have been discarded. The ‘Random’ points hold the baseline random performance values.

**Results.** Evaluation results when incrementally discarding samples from the paired training set, along with the random baseline performance results (labelled as Random) are presented in Table 6 and Figure 9. When considering the random performance baseline (i.e, Random column of Table 6), which averaged at 0.546 mAP for MIR-Flickr25K and 0.259 for NUS-WIDE, the baseline results are very close to those obtained when 100% of a given modality was unpaired (as shown in Table 3). When 100% of images or text were unpaired, average mAP scores of 0.564 for MIR-Flickr25K and 0.268 for NUS-WIDE were obtained (as shown in Table 3). As such, training with 100% of a modality being unpaired results in insignificant performance improvement (+0.014/+0.009 mAP for MIR-Flickr25K/NUS-WIDE) over the performance baseline.

In terms of sample discarding, DADH and AGAH benefit noticeably from additional training data as shown in Figure 9, showing a steady decrease in performance the more samples are discarded from the training set. JDSH remains more consistent in its performance relative to the amount of training samples being discarded. This suggests that DADH and AGAH would benefit from additional sources of training data, e.g., unpaired samples, while JDSH retains its performance even with reduced amounts of data. To investigate further, we next compare the sample discarding results to previously discussed unpaired sample training results.

Table 7 compares results previously obtained when training with four different training sets, each containing: unpaired images (UI), unpaired text (UT), both unpaired images and text (UIT) and sample discarding (SD). The results for the (i→t) and (t→i) tasks are compared separately and jointly. Table 7 shows which of the four cases (i.e., UI, UT, UIT, SD) resulted in the best retrieval performance. The percentage shown in the brackets is the performance difference by which a given unpaired sample case outperformed sample discarding. For example, in Table 7, when DADH is used for the (i→t) task, using 20% of Unpaired Text (UT) improved retrieval performance by 0.86% compared to when discarding 20% of samples (SD). The actual values can be seen in Table 4 and Table 6, where mAP was 0.824 mAP for SD and 0.831 mAP, respectively.

Although the DADH, AGAH and JDSH methods were not developed for unpaired training, using the UMML framework, training with unpaired samples resulted in improved results in 53 of the 90 cases evaluated. The performance improvement when including unpaired samples in the training set when compared to discarding the samples outright was more substantial the more limited the amount of paired data was available in the dataset. Therefore, strategies for adapting CMH methods to efficiently train with unpaired samples are needed, particularly when limited paired data is available and unpaired data need to be utilised for improving the learning of the model.

### 5.5. Comparison to Other Unpaired CMH Methods

The following is a comparison between the pairwise-constrained methods: DADH, AGAH and JDSH when using the proposed UMML framework to enable these methods to learn from fully unpaired samples; and the unpaired CMH methods: Adaptive Marginalized Semantic Hashing (AMSH) [43], Robust Unsupervised Cross-modal Hashing (RUCMH) [42] and Flexible Cross-Modal Hashing (FlexCMH) [44] that can learn from unpaired datasets. These experiments were conducted using the MIR-Flickr25K and NUS-WIDE datasets.

AMSH and FlexCMH are supervised methods that shuffle the training data to create unpaired sample behaviour. RUCMH is an unsupervised method that is independent of pairwise relationships. For the methods DADH, AGAH and JDSH, the 50% image and 50% text UMML unpairing approach is used as discussed in Section 5.3, where half of the text samples are emptied leaving 50% of the images being unpaired, and the other half of image samples are emptied leaving 50% of text being unpaired.

For AMSH, RUCMH and FlexCMH, the results in their respective publications are used for this comparison because the source-code for these methods are not publicly available. For a full specification regarding the training parameters of AMSH [43], RUCMH [42] and FlexCMH [44], please refer to their respective publications.

As shown in Table 8 AMSH outperforms the other methods by a considerable margin on the (t→i) task, with a 10.23% performance increase over the second best performing method, DADH + UMML. For the (t→i) task however, DADH + UMML narrowly obtains the best performance. The results obtained by the methods using the UMML extension are important because the methods are not designed for the unpaired scenario; the UMML approach adapts the datasets to the methods for these to be compatible with unpaired data. This indicates there is a need for approaches that fully adapt pairwise-constrained methods to the unpaired scenario.

### 5.6. Class-by-Class Performance Evaluations

The objective of this experiment is to evaluate the performance of each class in MIR-Flickr25K when training with unpaired samples using DADH. Evaluations were made on a class-by-class basis across the 24 classes in the dataset, where three training set pairing scenarios were evaluated: fully paired, 80% of the training text being unpaired, and 80% of the training images being unpaired.

Table 9 shows the results of the experiments. The first column, ‘MIR-Flick25 Classes’, shows the class number and name. The brackets next to each class indicate the number of queries taken from the given class and the possible number of correct retrievals within the retrieval set (Queries/Relevant Files). The column ‘Performance Difference’ shows the performance difference of the unpaired scenarios when compared to the paired scenario, with the values being computed using Formula (4).

**Figure 10:**
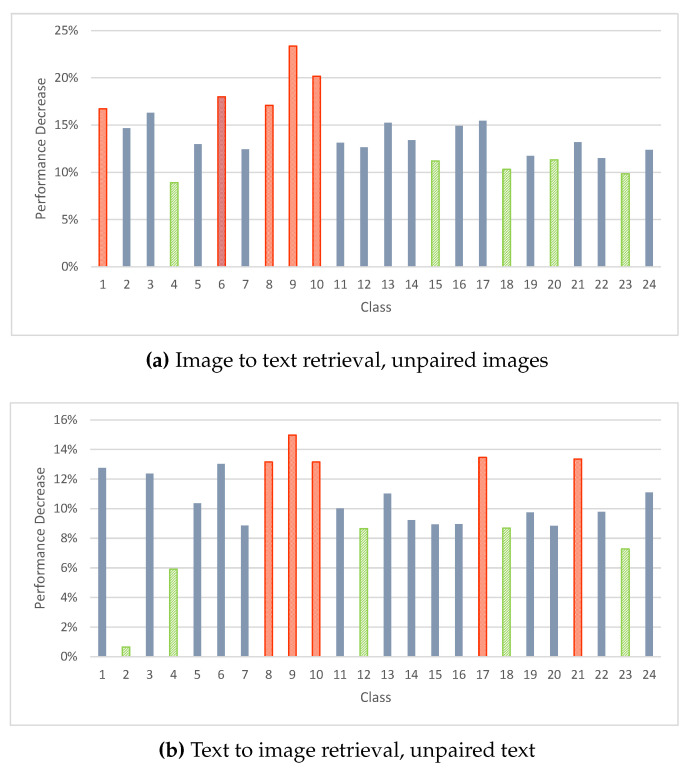
Percentage of performance change of DADH computed using formula (4) when training with unpaired samples compared to paired training across 24 classes of MIR-Flickr25K. Red bars show the five classes with the most performance change and green bars show the five classes with the least performance change. The remaining classes are marked as blue bars.

Figure 10 shows the performance difference across the classes when training with unpaired samples compared to when training with paired samples, calculated using formula (4). The performance difference across all classes was negative, meaning that the retrieval performance of the the DADH model was always worse when training with unpaired samples. The ‘Performance Decrease’ values shown on the y-axes of the charts of Figure 10, indicate a noticeable variation in performance difference across the classes when training with unpaired samples compared to training with paired samples. For example, classes 8, 9, and 10 were among the five most negatively affected classes when training with both unpaired images and text averaging a performance decrease of 16.98%. On the other hand, classes 4, 18 and 23 were among the five least negatively affected classes averaging a performance decrease of 8.49%. This variation found in the results across classes indicates that the type of data used impacts the degree to which performance is affected when adapting from training with paired data to training with unpaired data. As such, the content used for a given unpaired information retrieval task should be considered when developing strategies to tackle the unpaired scenario.

## 6. Conclusions

This paper explores the topic of Unpaired Cross-Modal Hashing (CMH) and the capabilities of state-of-the-art CMH methods with regards to learning from unpaired data in the context of information retrieval. The UMML framework has been proposed to enable pairwise-constrained CMH methods to learn from unpaired data. Through UMML, experiments have been conducted using DADH, AGAH and JDSH with paired and unpaired sample variations of MIR-Flickr25K and NUS-WIDE datasets. Evaluations to determine how unpaired data affect performance were carried out. Below is a summary of the main observations:

– Unpaired data can improve the training results of CMH methods. Furthermore, if data from both the image and text modalities are present in the training set, initially pairwise-constrained CMH methods can be trained on fully unpaired data.
– The extent to which unpaired data are helpful to the training process is relative to the amount of paired samples. The more scarce the paired samples available, the more helpful it can be to use additional unpaired samples for training.
– The performance of the models showcased when using unpaired samples for training is dependent on the modality of the unpaired samples, the dataset being used, the class of the unpaired data, and the architecture of the CMH algorithms. These factors influence whether unpaired samples will be helpful to the training process.
– The proposed UMML framework adapts the dataset to enable pairwise-constrained CMH methods to train on unpaired samples. When using UMML to enable DADH, AGAH and JDSH to train with unpaired samples, it was observed that the methods perform well when training with unpaired samples. This suggests that further improvements may be observed if the architectures of these methods are adapted to train on unpaired data.

With the findings obtained in this study, future works include extending the proposed UMML framework to adapt methods to training with unpaired samples at an architectural level. Future work also includes applying the UMML framework using data from case studies provided by industry.

## Figures and Tables

**Table 1:** MIRFlickr-25K and NUS-Wide dataset characteristics.

Dataset	Train	Query	Retrieval
MIRFlickr-25K	10,000	2000	18,015
NUS-Wide	10,000	2100	193,734

**Table 2:** Example of images, paired tags, and labels from the MIR-Flickr25K and NUS-WIDE datasets. Example images (1) and (2) reprinted under Creative Commons attribution, (1) Author: Martin P. Szymczak, Source, CC BY-NC-ND 2.0 (2) Title: Squirrel, Author: likeaduck, Source, CC BY 2.0.

Image	Tag	Label/Class
MIR-Flickr25K example (1)		
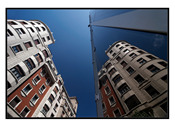	bilbao, 11–16, cielo, sky, polarizado, reflejo, reflection, sanidad, estrenandoMiRegalito, geotagged, geo:lat = 43.260867, geo:lon = −2.935705,	clouds, sky, structures
NUS-Wide example (2)		
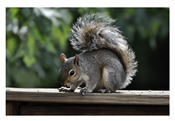	cute, nature, squirrel, funny, boxer, boxing, cuteness, coolest, pugnacious, peopleschoice, naturesfinest, blueribbonwinner, animalkingdomelite, mywinners, abigfave, superaplus aplusphoto, vimalvinayan, natureoutpost	Animal, Nature

**Table 3:** Results (mAP) on MIR-Flickr25K and NUS-WIDE with unpaired images, i.e., images with no corresponding text. Column ‘Paired’ shows results when training with a fully paired training set. Subsequent columns show results with increasing amounts of unpaired images in the training set.

		MIR-Flickr25K						NUS-WIDE					
**Task**	**Method**	**Paired**	**20%**	**40%**	**60%**	**80%**	**100%**	**Paired**	**20%**	**40%**	**60%**	**80%**	**100%**
i→t	DADH	0.836	0.807	0.789	0.750	0.702	0.562	0.701	0.690	0.683	0.656	0.646	0.297
	AGAH	0.803	0.752	0.729	0.695	0.637	0.535	0.633	0.621	0.583	0.587	0.503	0.267
	JDSH	0.672	0.653	0.648	0.643	0.619	0.555	0.546	0.534	0.510	0.457	0.402	0.253
** t→i **	DADH	0.823	0.824	0.814	0.812	0.796	0.552	0.707	0.706	0.702	0.670	0.634	0.261
	AGAH	0.790	0.790	0.786	0.779	0.742	0.540	0.646	0.595	0.591	0.596	0.401	0.277
	JDSH	0.660	0.672	0.666	0.652	0.632	0.564	0.566	0.499	0.476	0.452	0.412	0.256

**Table 4:** Results (mAP) on MIR-Flickr25K and NUS-WIDE with unpaired text, i.e., text with no corresponding images. Column ‘Paired’ shows results when training with a fully paired training set. Subsequent columns show results with increasing amounts of unpaired text in the training set.

		MIR-Flickr25K						NUS-WIDE					
**Task**	**Method**	**Paired**	**20%**	**40%**	**60%**	**80%**	**100%**	**Paired**	**20%**	**40%**	**60%**	**80%**	**100%**
i→t	DADH	0.836	0.831	0.831	0.826	0.820	0.525	0.701	0.700	0.696	0.683	0.674	0.282
	AGAH	0.803	0.755	0.740	0.720	0.682	0.541	0.633	0.597	0.566	0.500	0.356	0.267
	JDSH	0.672	0.646	0.621	0.608	0.580	0.553	0.546	0.515	0.478	0.393	0.342	0.254
t→i	DADH	0.823	0.803	0.783	0.756	0.711	0.545	0.707	0.705	0.724	0.697	0.698	0.274
	AGAH	0.790	0.760	0.744	0.698	0.642	0.535	0.646	0.645	0.653	0.651	0.464	0.267
	JDSH	0.660	0.653	0.622	0.631	0.601	0.545	0.566	0.520	0.506	0.468	0.420	0.249

**Table 5:** Results (mAP) on MIR-Flickr25K and NUS-WIDE with unpaired images and text, i.e., images with no corresponding text and vice versa. Column ‘Paired’ shows results when training with a fully paired training set. Subsequent columns show results with increasing amounts of unpaired images and text in the training set, for example, ‘10% 10%’ refers to 10% of the training set being unpaired images (UI) and another 10% being unpaired text (UT) for a total of 20% of the dataset being unpaired samples.

		MIR-Flickr25K							NUS-WIDE						
**Task**	**Method**	**Paired**	**UI:** **UT:**	**10%** **10%**	**20%** **20%**	**30%** **30%**	**40%** **40%**	**50%** **50%**	**Paired**	**UI:** **UT:**	**10%** **10%**	**20%** **20%**	**30%** **30%**	**40%** **40%**	**50%** **50%**
i→t	DADH	0.836		0.820	0.822	0.752	0.728	0.760	0.701		0.696	0.676	0.663	0.676	0.662
	AGAH	0.803		0.741	0.737	0.664	0.673	0.693	0.633		0.642	0.637	0.561	0.564	0.567
	JDSH	0.672		0.652	0.643	0.609	0.610	0.591	0.546		0.547	0.503	0.398	0.306	0.259
t→i	DADH	0.823		0.808	0.801	0.763	0.773	0.762	0.707		0.694	0.716	0.704	0.703	0.698
	AGAH	0.790		0.771	0.762	0.745	0.735	0.729	0.646		0.666	0.642	0.597	0.560	0.565
	JDSH	0.660		0.654	0.650	0.609	0.617	0.594	0.566		0.526	0.498	0.438	0.349	0.255

**Table 6:** Results (mAP) on MIR-Flickr25K and NUS-WIDE with sample discarding, i.e., training set being reduced. Column ‘Full’ shows results when training with full training set without any sample discarding. Subsequent columns show results with decreasing amounts of samples, where the given percentage denotes the percentage of samples in the training set which have been discarded. The ‘Random’ column holds the baseline random performance values.

		MIR-Flickr25K						NUS-WIDE					
**Task**	**Method**	**Full**	**20%**	**40%**	**60%**	**80%**	**Random**	**Full**	**20%**	**40%**	**60%**	**80%**	**Random**
i→t	DADH	0.836	0.824	0.799	0.779	0.744	0.543	0.701	0.683	0.648	0.610	0.575	0.260
	AGAH	0.803	0.763	0.737	0.714	0.678	0.548	0.633	0.633	0.588	0.440	0.366	0.267
	JDSH	0.672	0.657	0.655	0.640	0.634	0.551	0.546	0.543	0.523	0.469	0.457	0.256
** t→i **	DADH	0.823	0.807	0.797	0.781	0.754	0.537	0.707	0.672	0.663	0.630	0.549	0.258
	AGAH	0.790	0.779	0.778	0.756	0.730	0.538	0.646	0.567	0.547	0.488	0.377	0.267
	JDSH	0.660	0.669	0.654	0.654	0.644	0.559	0.566	0.514	0.517	0.487	0.424	0.245

**Table 7:** The sampling cases that produced the best retrieval results are indicated by UI: Unpaired Image, UT: Unpaired Text, UIT: Unpaired Image and Text, and SD: Sample discarding. The percentage shown in the brackets is the performance difference by which a given unpaired sample case (shown in Table 3, Table 4 and Table 5) outperformed sample discarding (SD) (shown in Table 6). The first row shows the percentage of training samples being unpaired (UI, UT, UIT), or discarded (SD) depending on the cell value.

		MIR-Flickr25K				
Task	Method	20%	40%	60%	80%	100%
i→t	DADH	**UT (+0.86%)**	**UT (+3.97%)**	**UT (+6.02%)**	**UT (+10.16%)**	**UIT (+39.93%)**
	AGAH	SD	**UT (+0.35%)**	**UT (+0.87%)**	**UT (+0.58%)**	**UIT (+26.43%)**
	JDSH	SD	SD	SD	SD	**UIT (+7.26%)**
t→i	DADH	**UI (+2.02%)**	**UI (+2.16%)**	**UI (+4.04%)**	**UI (+5.57%)**	**UIT (+41.93%)**
	AGAH	**UI (+1.52%)**	**UI (+0.95%)**	**UI (+2.98%)**	**UI (+1.67%)**	**UIT (+35.5%)**
	JDSH	**UI (+0.45%)**	**UI (+1.83%)**	SD	SD	**UIT (+6.26%)**
Both Tasks	DADH	**UT (+0.19%)**	**UIT (+1.74%)**	SD	**UT (+2.20%)**	**UIT (+40.93%)**
	AGAH	SD	SD	**UI (+0.24%)**	SD	**UIT (+30.92%)**
	JDSH	SD	**UI (+0.38%)**	**UI (+0.08%)**	SD	**UIT (+6.76%)**
		**NUS-WIDE**				
Task	Method	20%	40%	60%	80%	100%
i→t	DADH	**UT (+2.52%)**	**UT (+7.49%)**	**UT (+11.98%)**	**UT (+17.12%)**	**UIT (+154.54%)**
	AGAH	**UIT (+1.36%)**	**UIT (+8.46%)**	**UI (+33.40%)**	**UIT (+54.17%)**	**UIT (+112.43%)**
	JDSH	SD	SD	SD	SD	SD
t→i	DADH	**UT (+5.09%)**	**UT (+9.15%)**	**UIT (+11.65%)**	**UIT (+28.05%)**	**UIT (+170.35%)**
	AGAH	**UIT (+17.58%)**	**UT (+17.19%)**	**UT (+26.61%)**	**UT (+52.36%)**	**UIT (+111.42%)**
	JDSH	**UT (+1.17%)**	SD	SD	SD	SD
Both Tasks	DADH	**UT (+3.70%)**	**UT (+8.33%)**	**UT (+11.25%)**	**UIT (+22.67%)**	**UIT (+162.41%)**
	AGAH	**UIT (+9.02%)**	**UIT (+12.75%)**	**UI (+27.49%)**	**UIT (+51.35%)**	**UIT (+111.92%)**
	JDSH	SD	SD	SD	SD	SD

**Table 8:** Fully unpaired 64-Bit mAP evaluation results for unpaired CMH methods and traditional CMH methods using UMML.

Fully Unpaired	MIR-Flickr25K		NUS-WIDE	
	i→t	t→i	i→t	t→i
AMSH [43]	0.758	**0.840**	0.657	**0.805**
RUCMH [42]	0.719	0.732	0.650	0.657
FlexCMH [44]	0.572	0.568	0.426	0.418
DADH + UMML	**0.760**	0.762	**0.662**	0.698
AGAH + UMML	0.693	0.729	0.567	0.565
JDSH + UMML	0.591	0.594	0.259	0.255

**Table 9:** MIR-Flickr25K class-by-class mAP@N evaluation using 64-Bit DADH. Fully paired, 80% unpaired images and 80% unpaired text.

MIR-Flickr25K Classes	mAP						Performance Difference			
	Paired		Image Unpair		Text Unpair		Image Unpair		Text Unpair	
	i→t	t→i	i→t	t→i	i→t	t→i	i→t	t→i	i→t	t→i
1-Animals (271/2308)	0.777	0.744	0.647	0.723	0.779	0.649	−16.73%	−2.89%	0.21%	−12.76%
2-Baby (17/168)	0.881	0.815	0.752	0.866	0.897	0.809	−14.68%	6.32%	1.73%	−0.65%
3-Bird (63/552)	0.780	0.764	0.653	0.745	0.770	0.670	−16.29%	−2.56%	−1.32%	−12.37%
4-Car (90/926)	0.879	0.869	0.800	0.861	0.896	0.818	−8.90%	−0.96%	1.99%	−5.91%
5-Clouds (364/2883)	0.901	0.906	0.784	0.859	0.897	0.812	−12.98%	−5.24%	−0.38%	−10.37%
6-Dog (58/508)	0.791	0.755	0.648	0.714	0.785	0.656	−17.99%	−5.44%	−0.76%	−13.02%
7-Female (433/4243)	0.894	0.879	0.783	0.863	0.892	0.801	−12.43%	−1.79%	−0.20%	−8.87%
8-Flower (223/1273)	0.834	0.866	0.692	0.828	0.834	0.752	−17.09%	−4.41%	−0.11%	−13.16%
9-Food (73/747)	0.734	0.692	0.562	0.707	0.760	0.589	−23.36%	2.12%	3.53%	−14.97%
10-Indoor (550/5899)	0.836	0.791	0.667	0.795	0.844	0.687	−20.18%	0.61%	0.98%	−13.15%
11-Lake (27/609)	0.873	0.866	0.758	0.836	0.879	0.779	−13.14%	−3.54%	0.65%	−10.03%
12-Male (447/4375)	0.899	0.878	0.785	0.862	0.886	0.802	−12.64%	−1.76%	−1.39%	−8.66%
13-Night (227/2078)	0.850	0.841	0.720	0.820	0.836	0.749	−15.24%	−2.57%	−1.57%	−11.01%
14-People (769/7227)	0.892	0.872	0.772	0.858	0.885	0.792	−13.39%	−1.70%	−0.81%	−9.24%
15-$Plant_{l}ife$ (728/6535)	0.870	0.881	0.773	0.833	0.878	0.802	−11.20%	−5.37%	0.83%	−8.94%
16-Portrait (292/2524)	0.890	0.860	0.757	0.867	0.890	0.783	−14.91%	0.79%	0.06%	−8.97%
17-River (43/701)	0.885	0.883	0.748	0.829	0.862	0.765	−15.44%	−6.17%	−2.63%	−13.46%
18-Sea (87/961)	0.848	0.843	0.761	0.809	0.877	0.769	−10.31%	−3.99%	3.43%	−8.69%
19-Sky (639/6020)	0.895	0.900	0.790	0.851	0.891	0.812	−11.74%	−5.41%	−0.43%	−9.75%
20-Structures (779/7626)	0.888	0.884	0.787	0.849	0.887	0.806	−11.33%	−3.91%	−0.11%	−8.84%
21-Sunset (215/1696)	0.884	0.914	0.768	0.850	0.883	0.792	−13.20%	−7.07%	−0.09%	−13.36%
22-Transport (201/2219)	0.877	0.875	0.777	0.820	0.878	0.790	−11.48%	−6.28%	0.11%	−9.78%
23-Tree (342/3564)	0.899	0.901	0.810	0.857	0.910	0.835	−9.85%	−4.83%	1.29%	−7.28%
24-Water (271/2472)	0.837	0.839	0.733	0.793	0.845	0.746	−12.39%	−5.48%	1.00%	−11.10%

## Data Availability

The code for the experiments presented in this paper can be found in the project’s GitHub repository https://github.com/MikelWL/UMML (accessed on 9 December 2022). Publicly available datasets were analyzed in this study. This data can be found here: MIRFlickr25K: https://press.liacs.nl/mirflickr/ (accessed on 9 December 2022), NUS-WIDE: https://lms.comp.nus.edu.sg/wp-content/uploads/2019/research/nuswide/NUS-WIDE.html (accessed on 9 December 2022).

## References

[1] Baeza-Yates R., Ribeiro-Neto B. (1999). Modern Information Retrieval. Modern Information Retrieval.

[2] Lu X., Zhu L., Cheng Z., Song X., Zhang H. (2019). Efficient Discrete Latent Semantic Hashing for Scalable Cross-Modal Retrieval. Signal Process..

[3] Jin L., Li K., Li Z., Xiao F., Qi G.J., Tang J. (2018). Deep Semantic-Preserving Ordinal Hashing for Cross-Modal Similarity Search. IEEE Trans. Neural Netw. Learn. Syst..

[4] Kumar S., Udupa R. Learning Hash Functions for Cross-view Similarity Search. Proceedings of the 22nd International Joint Conference on Artificial Intelligence.

[5] Zhang D., Li W.J. Large-Scale Supervised Multimodal Hashing With Semantic Correlation Maximization. Proceedings of the AAAI Conference on Artificial Intelligence.

[6] Wang J., Zhang T., Sebe N., Shen H.T. (2017). A Survey on Learning to Hash. IEEE Trans. Pattern Anal. Mach. Intell..

[7] Deng C., Yang E., Liu T., Tao D. (2019). Two-Stream Deep Hashing with Class-Specific Centers for Supervised Image Search. IEEE Trans. Neural Netw. Learn. Syst..

[8] Peng Y., Huang X., Zhao Y. (2017). An Overview of Cross-Media Retrieval: Concepts, methodologies, Benchmarks, and Challenges. IEEE Trans. Circuits Syst. Video Technol..

[9] Jiang Q.Y., Li W.J. Deep Cross-Modal Hashing. Proceedings of the IEEE Conference on Computer Vision and Pattern Recognition.

[10] Gu W., Gu X., Gu J., Li B., Xiong Z., Wang W. Adversary Guided Asymmetric Hashing for Cross-Modal Retrieval. Proceedings of the 2019 on International Conference on Multimedia Retrieval.

[11] Liu S., Qian S., Guan Y., Zhan J., Ying L. Joint-Modal Distribution-Based Similarity Hashing for Large-Scale Unsupervised Deep Cross-Modal Retrieval. Proceedings of the 43rd International ACM SIGIR Conference on Research and Development in Information Retrieval.

[12] Bai C., Zeng C., Ma Q., Zhang J., Chen S. Deep Adversarial Discrete Hashing for Cross-Modal Retrieval. Proceedings of the 2020 International Conference on Multimedia Retrieval.

[13] Zheng L., Yang Y., Tian Q. (2017). SIFT Meets CNN: A Decade Survey of Instance Retrieval. IEEE Trans. Pattern Anal. Mach. Intell..

[14] Wang J., Liu W., Kumar S., Chang S.F. (2015). Learning to Hash for Indexing Big Data—A Survey. Proc. IEEE.

[15] Shen H.T., Liu L., Yang Y., Xu X., Huang Z., Shen F., Hong R. (2020). Exploiting Subspace Relation in Semantic Labels for Cross-Modal Hashing. IEEE Trans. Knowl. Data Eng..

[16] Ding K., Huo C., Fan B., Xiang S., Pan C. (2016). In Defense of Locality-Sensitive Hashing. IEEE Trans. Neural Netw. Learn. Syst..

[17] Hardoon D.R., Szedmak S., Shawe-Taylor J. (2004). Canonical Correlation Analysis: An Overview with Application to Learning Methods. Neural Comput..

[18] Liu X., Hu Z., Ling H., Cheung Y.M. (2019). MTFH: A Matrix Tri-Factorization Hashing Framework for Efficient Cross-Modal Retrieval. IEEE Trans. Pattern Anal. Mach. Intell..

[19] Cao W., Feng W., Lin Q., Cao G., He Z. (2020). A Review of Hashing Methods for Multimodal Retrieval. IEEE Access.

[20] Pereira J.C., Coviello E., Doyle G., Rasiwasia N., Lanckriet G.R., Levy R., Vasconcelos N. (2013). On the Role of Correlation and Abstraction in Cross-Modal Multimedia Retrieval. IEEE Trans. Pattern Anal. Mach. Intell..

[21] Young P., Lai A., Hodosh M., Hockenmaier J. (2014). From Image Descriptions to Visual Denotations: New Similarity Metrics for Semantic Inference Over Event Descriptions. Trans. Assoc. Comput. Linguist..

[22] Simonyan K., Zisserman A. Very Deep Convolutional Networks for Large-Scale Image Recognition. Proceedings of the International Conference on Learning Representations.

[23] Luo X., Wang H., Wu D., Chen C., Deng M., Huang J., Hua X.S. (2022). A Survey on Deep Hashing Methods. Acm Trans. Knowl. Discov. Data.

[24] Strecha C., Bronstein A., Bronstein M., Fua P. (2011). LDAHash: Improved Matching with Smaller Descriptors. IEEE Trans. Pattern Anal. Mach. Intell..

[25] He J., Liu W., Chang S.F. Scalable Similarity Search with Optimized Kernel Hashing. Proceedings of the 16th ACM SIGKDD International Conference on Knowledge Discovery and Data Mining.

[26] Gui J., Liu T., Sun Z., Tao D., Tan T. (2016). Supervised Discrete Hashing with Relaxation. IEEE Trans. Neural Netw. Learn. Syst..

[27] Gionis A., Indyk P., Motwani R. (1999). Similarity Search in High Dimensions via Hashing. Very Large Data Bases.

[28] Zhu X., Huang Z., Shen H.T., Zhao X. Linear Cross-Modal Hashing for Efficient Multimedia Search. Proceedings of the 21st ACM International Conference on Multimedia.

[29] Ding G., Guo Y., Zhou J. Collective Matrix Factorization Hashing for Multimodal Data. Proceedings of the IEEE Conference on Computer Vision and Pattern Recognition.

[30] Lin Z., Ding G., Han J., Wang J. (2016). Cross-View Retrieval via Probability-Based Semantics-Preserving Hashing. IEEE Trans. Cybern..

[31] Liu Q., Liu G., Li L., Yuan X.T., Wang M., Liu W. (2017). Reversed Spectral Hashing. IEEE Trans. Neural Netw. Learn. Syst..

[32] Liu X., Yu G., Domeniconi C., Wang J., Ren Y., Guo M. (2019). Ranking-Based Deep Cross-Modal Hashing. Proc. Aaai Conf. Artif. Intell..

[33] Wang J., Liu W., Sun A.X., Jiang Y.G. Learning Hash Codes with Listwise Supervision. Proceedings of the IEEE International Conference on Computer Vision.

[34] Jin Z., Hu Y., Lin Y., Zhang D., Lin S., Cai D., Li X. Complementary Projection Hashing. Proceedings of the IEEE International Conference on Computer Vision.

[35] Yang E., Deng C., Liu W., Liu X., Tao D., Gao X. Pairwise Relationship Guided Deep Hashing for Cross-Modal Retrieval. Proceedings of the AAAI Conference on Artificial Intelligence.

[36] Li C., Deng C., Li N., Liu W., Gao X., Tao D. Self-Supervised Adversarial Hashing Networks for Cross-Modal Retrieval. Proceedings of the IEEE Conference on Computer Vision and Pattern Recognition.

[37] Mandal D., Chaudhury K.N., Biswas S. Generalized Semantic Preserving Hashing for N-Label Cross-Modal Retrieval. Proceedings of the IEEE Conference on Computer Vision and Pattern Recognition.

[38] Hu Z., Liu X., Wang X., Cheung Y.m., Wang N., Chen Y. Triplet Fusion Network Hashing for Unpaired Cross-Modal Retrieval. Proceedings of the 2019 on International Conference on Multimedia Retrieval.

[39] Wen X., Han Z., Yin X., Liu Y.S. Adversarial Cross-Modal Retrieval via Learning and Transferring Single-Modal Similarities. Proceedings of the 2019 IEEE International Conference on Multimedia and Expo (ICME).

[40] Gao J., Zhang W., Zhong F., Chen Z. (2020). UCMH: Unpaired Cross-Modal Hashing with Matrix Factorization. Elsevier Neurocomput..

[41] Liu W., Wang J., Kumar S., Chang S. Hashing with graphs. Proceedings of the International Conference on Machine Learning.

[42] Cheng M., Jing L., Ng M. (2020). Robust Unsupervised Cross-modal Hashing for Multimedia Retrieval. ACM Trans. Inf. Syst. (TOIS).

[43] Luo K., Zhang C., Li H., Jia X., Chen C. (2022). Adaptive Marginalized Semantic Hashing for Unpaired Cross-Modal Retrieval. arXiv.

[44] Yu G., Liu X., Wang J., Domeniconi C., Zhang X. (2020). Flexible Cross-Modal Hashing. IEEE Trans. Neural Netw. Learn. Syst..

[45] Huiskes M.J., Lew M.S. The MIR Flickr Retrieval Evaluation. Proceedings of the 1st ACM International Conference on Multimedia Information Retrieval.

[46] Chua T.S., Tang J., Hong R., Li H., Luo Z., Zheng Y. NUS-WIDE: A Real-World Web Image Database From National University of Singapore. Proceedings of the ACM International Conference on Image and Video Retrieval.

